# Transcollateral retrograde recanalization of superior mesenteric artery occlusion through the pancreaticoduodenal arcade

**DOI:** 10.1016/j.jvscit.2024.101699

**Published:** 2024-11-30

**Authors:** Khaled El-Qawaqzeh, Romeo Mateo, Heepeel Chang, Arun Goyal, Sateesh Babu, Daniel J. Ventarola

**Affiliations:** Department of Surgery, Westchester Medical Center, Valhalla, NY

**Keywords:** Chronic mesentric ischemia, Endovascular stenting placement, Mesenteric stenting, Retrograde approach, Shockwave intravascular lithotripsy

## Abstract

We present a case of an 86-year-old female with chronic mesenteric ischemia secondary to long-segment flush occlusion of the superior mesenteric artery and near-total occlusion of the celiac artery. The superior mesenteric artery was unable to be revascularized by conventional antegrade approaches. Successful transcollateral crossing of the occluded superior mesenteric artery and body-flossing, followed by antegrade balloon angioplasty, shockwave lithotripsy, and stent implantation were performed. This case demonstrates that retrograde recanalization via collateral pathways is a viable alternative for patients with superior mesenteric artery flush occlusion when conventional antegrade approaches fail.

Chronic mesenteric ischemia (CMI) occurs secondary to visceral malperfusion, most commonly secondary to atherosclerotic disease.^1^ Typically, patients with CMI present with postprandial abdominal pain, food avoidance, and progressive weight loss.^2^ Treatment is indicated for all symptomatic cases and can be performed via both open and endovascular techniques. Open operative intervention may be associated with considerable postoperative morbidity and mortality. Advances in endovascular treatment, including balloon angioplasty and stent implantation, have made these techniques a preferred choice for CMI due to their lower morbidity, making them particularly suitable for these patients with often poor baseline conditions.^3^ We present a case of an 86-year-old female with multiple comorbidities who presented with progressive CMI with long segment flush occlusion of the superior mesenteric artery (SMA) and near-total occlusion of the celiac artery. She could not be revascularized through conventional antegrade endovascular methods. Utilizing the pancreaticoduodenal arcade (PDA) collateral pathway, successful retrograde recanalization, intravascular lithotripsy, and stent implantation were performed, resulting in the full resolution of her symptoms. This report demonstrates retrograde recanalization of the SMA as a feasible alternative when conventional anterograde approaches fail.

## Case report

This is an 86-year-old female with medical history significant for hypertension and hyperlipidemia, who was recently admitted with worsening of abdominal pain. She experienced intermittent postprandial episodes of abdominal pain over the past 6 months and reported persistent diarrhea. She also noted a weight loss of over 18 kg in the past 3 to 4 years, including a recent loss of 4.5 kg resulting in a body mass index of 16 kg/m^2^. Computed tomography angiogram was performed and demonstrated long-segment flush occlusion of SMA (length approximately 37 mm) with dense calcification and near total occlusion of the celiac artery. A robust PDA was also apparent. The patient was offered revascularization via angiogram with intention to treat. Given her advanced age, frailty, malnutrition, extreme weight loss, hypertension, and hyperlipidemia, she was felt to be a poor first-line open operative candidate. Additionally, a recent left shoulder fracture was preclusive of left arm supination and abduction for access, necessitating the use of the right arm.

**Fig 1:**
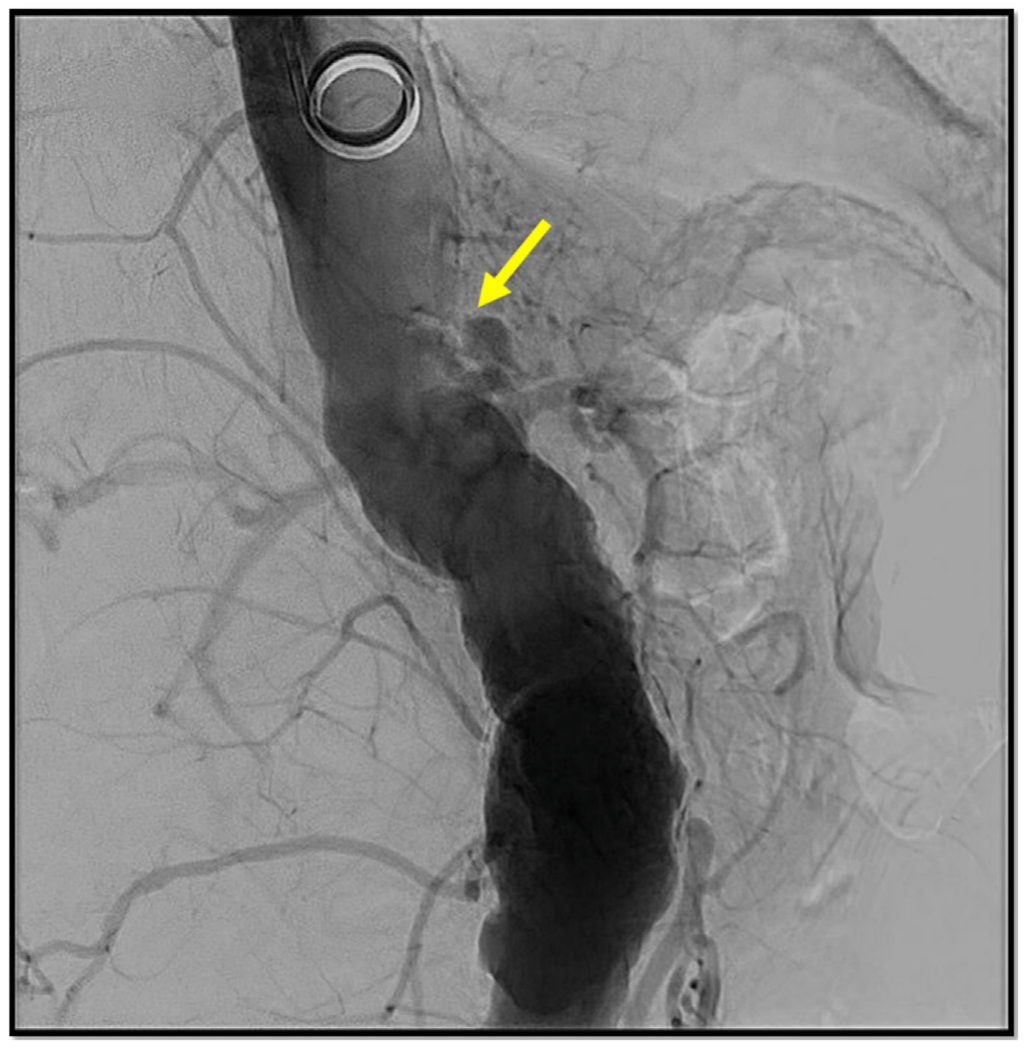
Aortogram showing featureless aorta with high grade celiac stenosis (*arrow*) and superior mesenteric artery (SMA) occlusion (origin of the SMA could not be seen in the anterograde approach, calcium deposit was noted inferior to the celiac origin, consistent with calcified SMA origin).

Open right brachial exposure was performed, and a 7-French sheath was advanced to the supraceliac level. Aortogram was performed, and although the celiac artery was visualized with high-grade stenosis, the SMA was not visualized due to flush occlusion (Fig 1). Attempts were made to probe the area of the presumed SMA orifice based on calcifications and other landmarks; however, these attempts were unsuccessful.

**Fig 2:**
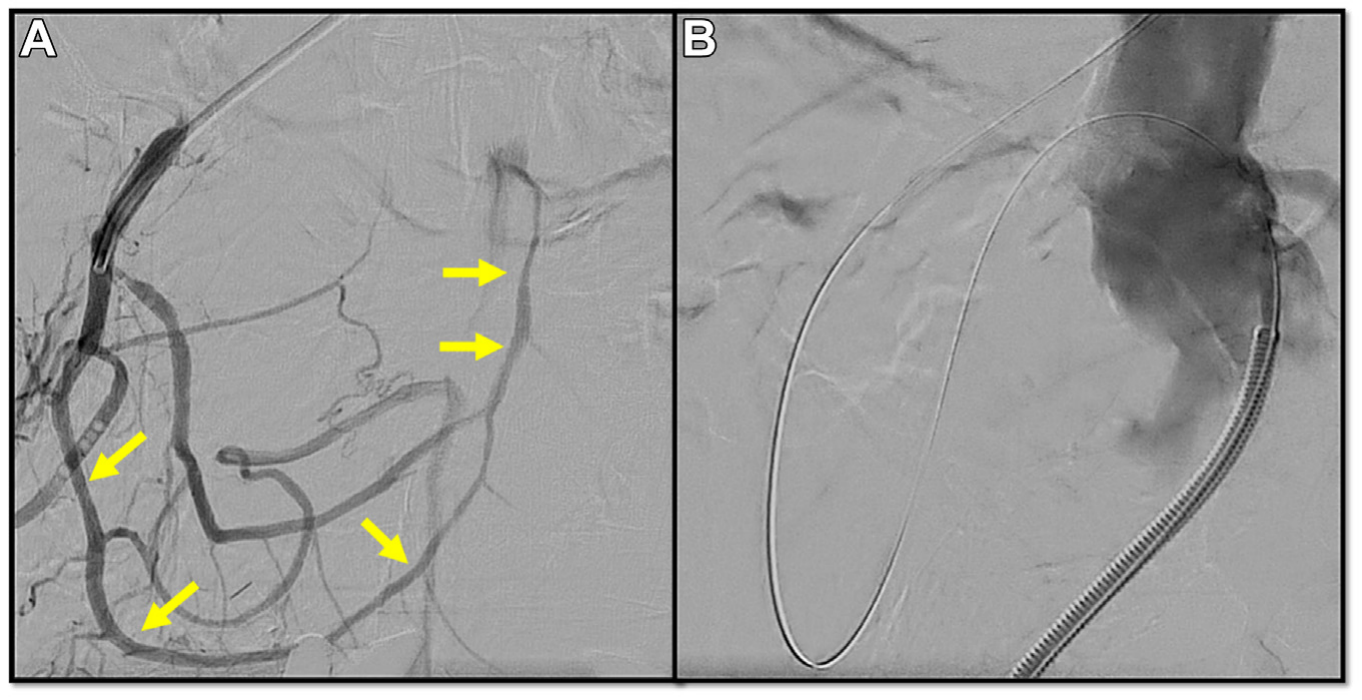
Visualization of pancreaticoduodenal arcade and reconstitution of the superior mesenteric artery (SMA) (*arrows point to the utilized collateral*) (anteroposterior) **(A)**. Though-and-though wire placement prior to intervention (lateral) **(B)**.

Thus, the celiac artery was then selected with a Glidewire and MPA catheter. Angiogram here delineated the takeoff of the gastroduodenal artery (GDA) and the PDA (Fig 2, *A*). We decided to attempt retrograde recanalization of the SMA. The GDA was selected with MPA as a parent catheter. We traversed the collateral with an atraumatic 0.014 wire, and then after confirming appropriate placement in the SMA, we switched it to weighted tip tapered coronary CTO wires used in conjunction with 0.014 coronary CTO microcatheter to traverse the arcade into the reconstituted segment of the SMA. By carefully manipulating the wire and avoiding inadvertent looping or knuckling of hydrophilic wires, which can lead to entering a subintimal plane, we successfully navigated the crossing and were able to advance into the aorta.

A 6-French sheath was placed via right groin access. The 0.014 through-wire was then snared via the right groin sheath and externalized (Fig 2, *B*). Now with through-and-through access, we first began with predilation of the SMA using a 4-mm balloon followed by a 5-mm lithotripsy balloon throughout the course of the occluded SMA. The angiogram now demonstrated an antegrade flow channel in the SMA. A 6 × 39 mm VBX stent had been advanced via right groin access and deployed, followed by an additional 6 × 19 mm stent distally. Care was taken to ensure these stents did not cover the origin of the inferior PDA.

**Fig 3:**
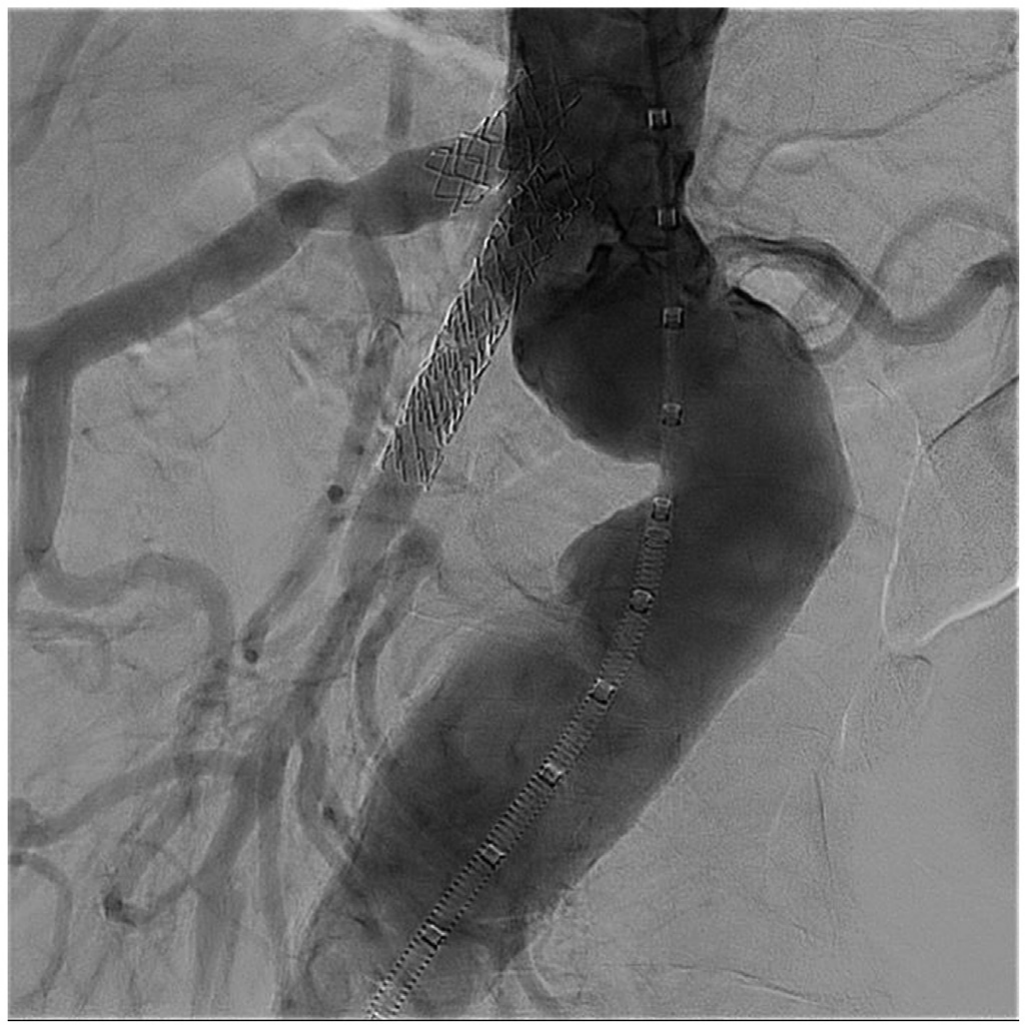
Completion angiogram after celiac and superior mesenteric artery (SMA) stenting.

At this point, we withdrew our through-and-through wire and turned our attention to the celiac axis. A 6 × 19 mm VBX stent was then deployed. A final angiogram was performed and demonstrated excellent flow now through the celiac axis, gastroduodenal arcade, and SMA, with excellent filling of SMA arcades (Fig 3).

The patient recovered well, with complete resolution of postprandial pain and restored oral intake. The patient was discharged 3 days after the procedure on dual antiplatelet therapy, and her improvement was maintained at the 1-month and 4-month follow-ups. In addition, postoperative abdominal ultrasound at 1-month and 4-month follow-ups showed widely patent celiac artery and SMA.

Publication consent was obtained from the patient.

## Discussion

CMI, or intestinal angina, involves intermittent or continuous reduced blood flow to the small intestine. This condition is usually most commonly due to atherosclerotic disease.^2^ Typically, patients with CMI present with postprandial abdominal pain, food avoidance, and progressive weight loss.^2^ Treatment for CMI is indicated for all cases of symptomatic intestinal ischemia. Open operative intervention may be associated with considerable postoperative morbidity and mortality, particularly in this patient population, who are often elderly, with significant weight loss, malnutrition, presence of coronary artery disease, hypertension, and other vascular occlusive diseases.^4^^,^^5^ Recent advances in endovascular treatment, including balloon angioplasty and stent implantation, have made these techniques a preferred choice for CMI due to their lower morbidity, making them particularly suitable for patients with poor baseline conditions.^3^

Given this 86-year-old patient’s advanced age, multiple comorbidities, and significant weight loss, she was deemed a poor candidate for open surgery. Percutaneous antegrade revascularization was initially attempted; however, conventional antegrade endovascular stenting is not feasible for all cases of CMI. When endovascular methods fail, an open surgical bypass is essential for restoring blood flow. Common bypass options include aortomesenteric, iliomesenteric, and aortoiliac bypass. Alternatively, retrograde open mesenteric stenting can be used, combining open surgical access with endovascular stenting, but this requires an open laparotomy.^6^ A potential alternative utilizes a retrograde crossing approach, which has been previously described in literature.^7^, ^8^, ^9^, ^10^, ^11^ In this case, the SMA origin was non-visualized due to a flush occlusion. In most cases, the collateral pathways linking the celiac trunk or inferior mesenteric artery with the distal SMA tend to open and enlarge as a compensatory mechanism.^12^ Therefore, we used an unconventional method involving retrograde crossing through the GDA and the PDA. This was followed by lithotripsy to improve compliance due to dense calcification, and finally with covered stenting.

This case report demonstrates that retrograde transcollateral recanalization of CMI is a feasible alternative for select patients with poor baseline status and mature collateral circulation when conventional approaches fail. Compared with open surgery, endovascular treatment offers several advantages, including less surgical stress, quicker recovery, shorter hospitalization, lower surgical risks, and fewer complications.^3^^,^^13^, ^14^, ^15^ Nevertheless, success of this method greatly hinges on the presence of a suitable collateral arch linking the celiac trunk/inferior mesenteric artery and the distal SMA. Thorough preoperative evaluation of images and intraoperative angiography can provide valuable insights to ascertain the retrograde recanalization technique’s feasibility. Additionally, several challenges to this approach should be acknowledged. First, the retrograde route is significantly long and tortuous, which can impede pushability of the catheter and guidewire. Second, the PDA is relatively small, fragile, and prone to injury. Furthermore, there is always a concern that the pull force generated during the snaring procedure might cause a cheese-cutting-like injury to the PDA, and thus catheter coverage should be maintained for as much of the paravisceral floss loop as possible.

## Conclusion

In conclusion, retrograde revascularization of chronic SMA occlusions via collateral vessels is feasible but technically challenging. For elderly patients with CMI who are at high risk for perioperative complications, endovascular strategies offer a viable option and may lead to sustained symptomatic improvement. It is sensible to consider this less-invasive method for treating older, high-risk patients who failed the conventional anterograde approach before considering open retrograde stenting or bypass.

## Funding

None.

## Disclosures

None.
